# Immune‐Induced Fever and Psychogenic Hyperthermia Can Occur Independently of Uncoupling Protein 1‐Mediated Brown Adipose Tissue Thermogenesis

**DOI:** 10.1111/apha.70300

**Published:** 2026-08-25

**Authors:** Takashi Matsuwaki, Wakana Harigai, Vladimir Maksimov, Yutaro Nakayama, Kazuyuki Uchida, James K. Chambers, Keitaro Yamanouchi, Anna Eskilsson, Anders Blomqvist

**Affiliations:** ^1^ Laboratory of Veterinary Physiology Graduate School of Agricultural and Life Sciences, The University of Tokyo Tokyo Japan; ^2^ Division of Neurobiology, Department of Biomedical and Clinical Sciences, Faculty of Medicine and Health Sciences Linköping University Linköping Sweden; ^3^ Laboratory of Veterinary Pathology Graduate School of Agricultural and Life Sciences, The University of Tokyo Tokyo Japan; ^4^ Clinical Rheumatology, Division of Inflammation and Infection, Department of Biomedical and Clinical Sciences, Faculty of Medicine and Health Sciences Linköping University Linköping Sweden

**Keywords:** brown adipose tissue, fever, psychogenic hyperthermia, rat, uncoupling protein 1

## Abstract

**Aim:**

Brown adipose tissue thermogenesis is widely considered an important effector mechanism in fever and emotionally induced hyperthermia (psychogenic fever), and increases in interscapular temperature are commonly interpreted as evidence of brown adipose tissue activation. Yet direct evidence for this interpretation remains limited and conflicting. Here, we tested these assumptions using complementary genetic and surgical approaches in rats.

**Methods:**

We generated uncoupling protein 1 knockout rats, in which canonical brown adipose tissue thermogenesis is abolished, and examined body temperature responses to systemic inflammation induced by lipopolysaccharide and to emotional stressors, including restraint and cage exchange. In separate experiments, interscapular brown adipose tissue was surgically removed in wild‐type rats.

**Results:**

Despite profound impairment of cold‐ and β_₃_‐adrenergic‐induced thermogenesis, uncoupling protein 1 deletion did not affect lipopolysaccharide‐ or stress‐induced increases in core or interscapular temperature. Likewise, removal of interscapular brown adipose tissue did not alter the interscapular temperature response. Thus, increases in interscapular temperature occurred in the absence of both uncoupling protein 1 and interscapular brown adipose tissue. Consistent with these findings, immune challenge and emotional stress induced little or no change in thermogenic gene expression and had minimal effects on tissue mass, in contrast to robust activation by β_₃_‐adrenergic stimulation.

**Conclusion:**

Fever and psychogenic hyperthermia in rats do not require uncoupling protein 1‐mediated brown adipose tissue thermogenesis. Furthermore, increases in interscapular temperature are not necessarily indicative of brown adipose tissue thermogenesis, challenging a widely held interpretation of these phenomena.

## 1 Introduction

While it is well established that UCP1‐mediated BAT thermogenesis is the principal mechanism of adaptive nonshivering thermogenesis during cold exposure [1], its role in fever and emotionally induced hyperthermia (psychogenic fever) remains disputed. Evidence from studies in rats has suggested that BAT thermogenesis is responsible for elevations in body temperature elicited by immune stimulation or emotional stress. For example, injection of prostaglandin E_2_, the final mediator of inflammation‐induced fever [2], into the thermoregulatory region of the preoptic hypothalamus not only raises body temperature but also increases sympathetic activity in nerve bundles at the surface of interscapular BAT pads [3, 4]. Such findings have been interpreted to suggest that the febrile response is mediated through sympathetic activation of BAT. Additional support for this interpretation comes from observations that temperature in the interscapular region rises before core body temperature following immune stimulation or during emotional stress [5, 6, 7], consistent with, but not definitive for, a causal role of BAT thermogenesis.

However, these lines of evidence are indirect. In contrast, functional studies in mice lacking uncoupling protein 1 (UCP1), the central mediator of BAT thermogenesis [1], have shown no impairment of fever or emotional stress‐induced hyperthermia [8, 9, 10, 11]. Moreover, immune stimulation or emotional stress does not induce upregulation of *Ucp1* or related thermogenic genes in wild‐type animals, in stark contrast to the robust induction observed during cold exposure or following administration of a β_₃_‐adrenergic agonist [11], both of which strongly activate BAT thermogenesis.

Despite these findings, the prevailing view that BAT thermogenesis contributes to fever and emotional hyperthermia has remained largely unchanged. One reason may be that most studies supporting a role for BAT have been performed in rats, whereas evidence against such a role has largely come from mice. Because rats and mice differ in thermoregulatory characteristics, including body size, surface‐to‐volume ratio, and the definition and range of the thermoneutral zone [12, 13], it has remained uncertain whether findings obtained in one species can be directly generalized to the other. Moreover, the increase in interscapular temperature, widely taken as evidence for BAT activation, has not been directly tested in relation to BAT thermogenesis. As a result, the concept that both immune‐induced fever and emotional hyperthermia depend on BAT thermogenesis remains influential and is often presented as established knowledge [14, 15].

In the present study, we directly addressed this issue using a purpose‐generated UCP1 knockout rat model and by surgically removing interscapular BAT in wild‐type rats. These complementary approaches allowed us to test both the requirement for UCP1 and the interpretation of the key observation underlying the prevailing view, namely the rapid increase in interscapular temperature during immune challenge and emotional stress. We show that this temperature increase persists both in UCP1‐deficient animals and following removal of interscapular BAT, demonstrating that the phenomenon widely taken as evidence for BAT activation occurs independently of BAT thermogenesis.

Together, these findings demonstrate that the peripheral thermogenic mechanisms underlying immune‐induced fever and emotional stress‐induced hyperthermia may differ from those mediating cold defense. Neither UCP1 nor interscapular BAT is required for increases in body temperature during immune or emotional stress, demonstrating that classical BAT‐mediated thermogenesis is not necessary for these responses.

## 2 Results

### 2.1 Characterization of UCP1 Knockout Rats

**FIGURE 1:**
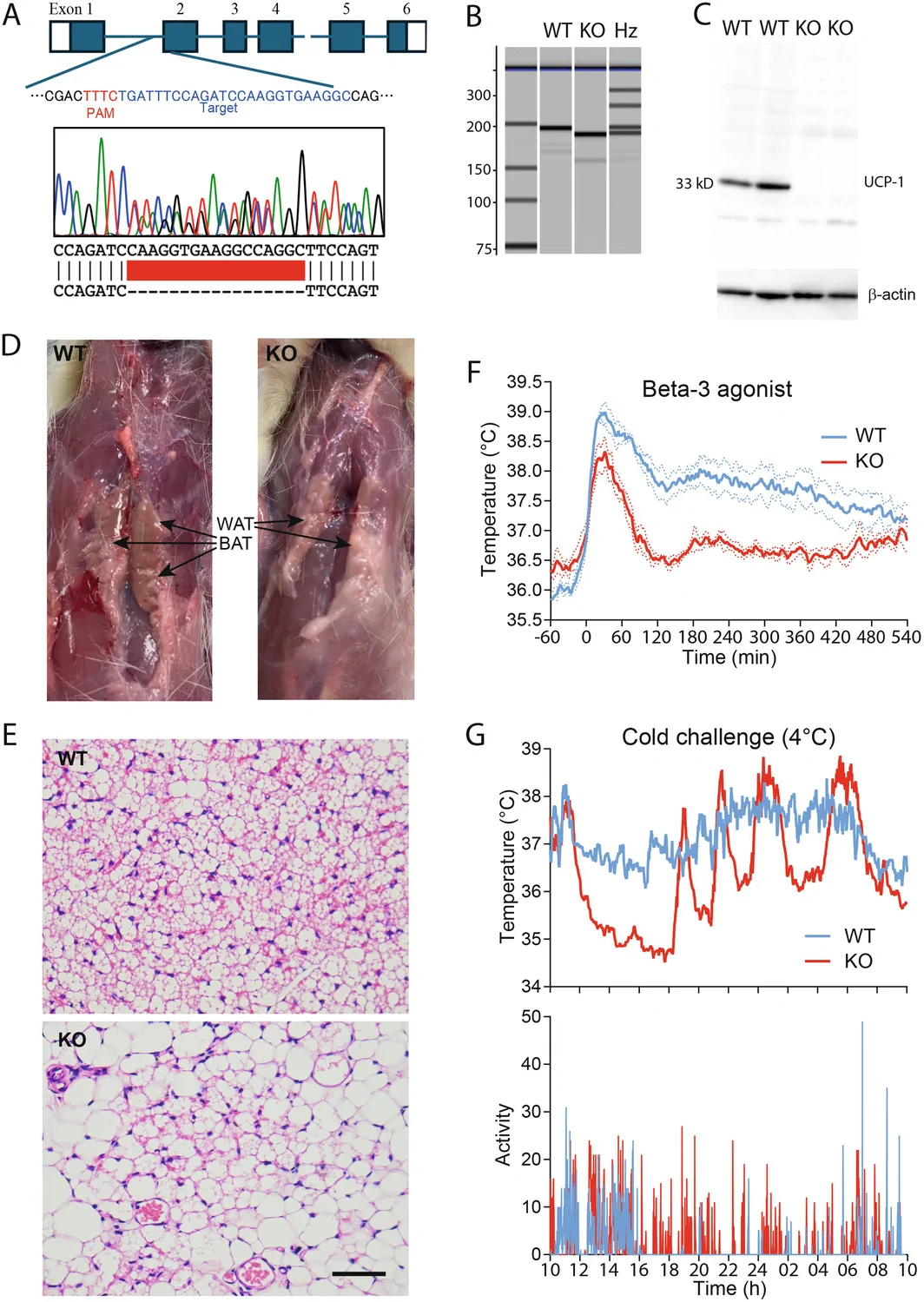
Generation and characterization of UCP1 knockout rats. (A) Strategy for targeted deletion of the *Ucp1* gene. Top: The target sequence (blue) was TGATTTCCAGATCCAAGGTGAAGGC, adjacent to a TTTC PAM site (red) in exon 2. Bottom: Site of the deletion (red bar) as determined by sequencing. (B) Microchip‐based capillary electrophoresis showing a 17‐bp deletion in the rat *Ucp1* gene; the two upshifted bands in heterozygous (Hz) rats represent heteroduplexes. (C) Western blot confirming the absence of UCP1 protein in UCP1 knockout (KO) rats. (D) Representative photographs of the interscapular region in WT and UCP1 KO rats. In WT rats, interscapular BAT was readily identified, whereas the corresponding region in UCP1 KO rats had a whitish appearance. (E) Hematoxylin–eosin staining of interscapular adipose tissue from a WT rat and the UCP1 KO rat in which residual BAT was observed, showing markedly enlarged lipid droplets in the KO. Scale bar, 50 μm. (F) A β_₃_‐adrenergic agonist induces sustained hyperthermia in WT but not UCP1 KO rats; the initial temperature peak reflects handling stress associated with injection at time 0. Solid lines show the mean, and dotted lines show the SEM. *n* = 4. (G) Exemplary data traces showing body temperature (upper) and locomotor activity (lower) in WT and UCP1 KO rats during exposure to 4°C (see also Figure S2).

Inactivation of the *Ucp1* gene, carried out in Wistar–Imamichi rats, was achieved by a targeted deletion in exon 2 (Figure 1A), using the i‐GONAD method [16] and the CRISPR/Cas12 system. The absence of *Ucp1* mRNA and UCP1 protein was confirmed by PCR and Western blot analyses, respectively (Figure 1B,C).

UCP1 knockout (KO) rats exhibited normal gross development. However, body weight in male KO rats was slightly lower than in wild‐type (WT) or heterozygous (Hz) rats, which did not differ from each other (Figure S1). Female KO rats showed a tendency toward lower body weight between 5 and 9 weeks of age, but this difference was no longer present by 11 weeks (Figure S1).

Adipose tissue in the interscapular region, where BAT is located in WT rats, appeared whitish in KO rats (Figure 1D). Histological analysis showed white adipose tissue in the interscapular region of four of five UCP1 KO rats. In the remaining animal, residual brown adipose tissue with markedly enlarged lipid droplets was observed (Figure 1E).

Administration of the β₃‐adrenergic agonist CL 316243 (1.0 mg/kg, i.p.), a potent inducer of BAT thermogenesis [17], elicited a similar initial handling stress‐induced hyperthermia in both WT and UCP1 KO rats. In WT rats, body temperature remained elevated following injection, whereas in KO rats it returned to pre‐injection levels (two‐way ANOVA for 120–540 min: *F*_1,6_ = 14.7, *p* = 0.0095), indicating that the β_₃_‐agonist failed to induce thermogenesis in the absence of UCP1 (Figure 1F).

Consistent with impaired BAT function, UCP1 KO rats exhibited abnormal cold defense. When exposed to an ambient temperature of 4°C, WT rats maintained a stable body temperature with a normal diurnal rhythm, characterized by slightly lower temperatures during the light (inactive) period than during the dark (active) period. In contrast, UCP1 KO rats displayed a lower and more variable baseline body temperature. Periods of increased locomotor activity were observed, corresponding to transient elevations in body temperature (Figure 1G; Figure S2).

### 2.2 Emotional Stress and Immune Challenge Induce Comparable Increases in Core and Interscapular Temperatures in UCP1 Knockout and Wild‐Type Rats

As shown in Figure 1F, UCP1 KO rats exhibited handling stress‐induced hyperthermia comparable to that observed in WT rats following intraperitoneal injection. We next examined thermoregulatory responses to emotional stress using two other paradigms: cage exchange and restraint stress. In separate experiments, we measured either intraperitoneal (core) temperature or temperature subcutaneously in the interscapular region overlying the BAT.

**FIGURE 2:**
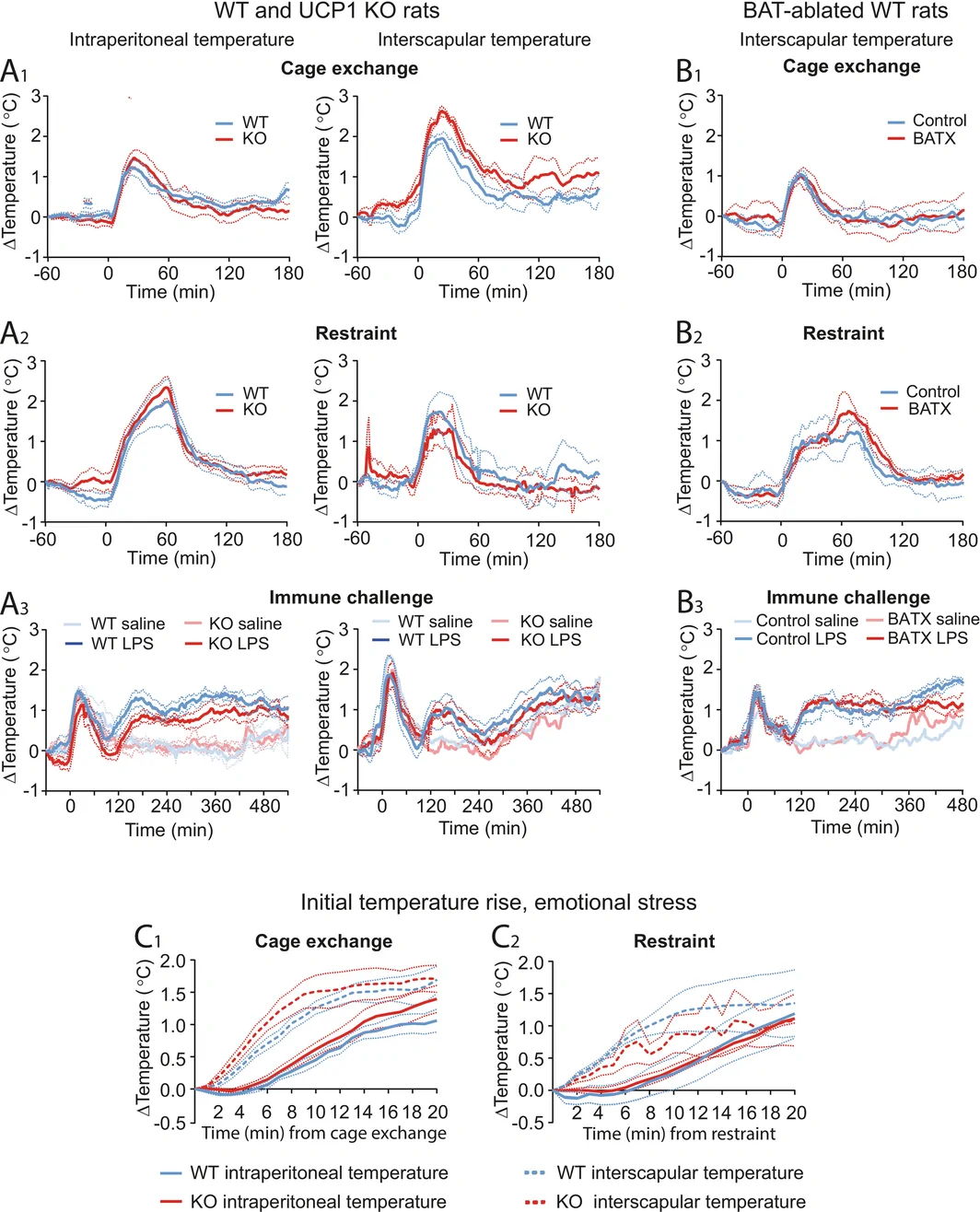
Temperature responses to emotional and immune stress. (A) Intraperitoneal (left) and interscapular (right) temperature recordings in WT and UCP1 knockout (KO) rats during emotional stress. (B) Interscapular temperature responses in sham‐operated WT rats (control) or WT rats following surgical removal of interscapular BAT (BATX). (A_1_, B_1_) Cage exchange. (A_2_, B_2_) Restraint. (A_3_, B_3_) Immune challenge with LPS. (C) Initial temperature rise during (C_1_) cage exchange and (C_2_) restraint. All interventions were performed at time point 0. Solid lines show the mean, and dotted lines show SEM. *n* = 4–5 (abdominal temperature) and 3–4 (interscapular temperature) in A_1_, A_2_, C_1_, and C_2_; *n* = 3–4 (abdominal temperature) and 6–8 (interscapular temperature) in A_3_; *n* = 4 in B_1_–B_3_. Detailed statistical analyses are provided in Tables S1 and S2.

Exposure to a cage previously occupied by another rat elicited pronounced hyperthermia in both UCP1 KO and WT rats (Figure 2A_1_). A similar increase in interscapular temperature was observed in both genotypes (Figure 2A_1_). Statistical analysis revealed minor genotype‐dependent differences in the temporal profile of the abdominal temperature response, but no overall genotype effect, whereas the interscapular temperature response was similar in WT and UCP1 KO rats (Table S1). Likewise, restraint stress induced by confinement in a plastic cylinder produced rapid increases in both intraperitoneal and interscapular temperature that were indistinguishable between UCP1 KO and WT rats (Figure 2A_2_; Table S1). Intraperitoneal administration of lipopolysaccharide (LPS), a well‐established model for peripheral inflammation and fever [18, 19], produced, after the initial similar handling stress‐induced hyperthermia, a febrile response in both WT and UCP1 KO rats (Figure 2A_3_). Similarly, an increase in interscapular temperature was observed following LPS administration in both genotypes (Figure 2A_3_). Statistical analysis revealed modest genotype‐dependent differences in the temporal profile of the abdominal temperature response, whereas neither the overall magnitude of the febrile response nor the interscapular temperature response differed between WT and UCP1 KO rats (Table S1). Together, these findings indicate that emotional stress‐induced and immune‐induced elevations in core body temperature and interscapular temperature can occur independently of UCP1‐mediated BAT thermogenesis.

We next compared the temporal dynamics of temperature changes intraperitoneally and in the interscapular region following emotional stress in WT and KO animals. After both cage exchange and restraint and irrespective of genotype, interscapular temperature increased within minutes, whereas the rise in intraperitoneal temperature was delayed by several minutes (Figure 2C_1,2_). The initial rate of temperature increase also tended to be steeper in the interscapular region than intraperitoneally (Figure 2C_1,2_), although this difference reached statistical significance only in wild‐type rats subjected to cage exchange (linear regression: *F*_1,76_ = 19.75, *p* < 0.001).

### 2.3 Removal of Interscapular BAT Does Not Affect Emotional Stress‐Induced or Immune‐Induced Increases in Interscapular Temperature

The findings above suggest that the rise in interscapular temperature following emotional stress or immune challenge is not mediated by UCP1‐dependent thermogenesis. To determine whether this temperature increase required the presence of interscapular BAT itself, and to exclude the possibility that UCP1‐independent thermogenic mechanisms within BAT contribute to this response [20], we performed a separate series of experiments in wild‐type rats in which the interscapular BAT was surgically removed. Temperature in the interscapular region was subsequently measured using a subcutaneously implanted transponder.

Exposure to a cage previously occupied by another rat elicited a marked increase in interscapular temperature that did not differ between sham‐operated and BATX rats (Figure 2B_1_). Likewise, restraint stress induced a robust increase in interscapular temperature of similar magnitude and time course in both groups (Figure 2B_2_). Intraperitoneal injection of LPS produced a clear increase in interscapular temperature, and the response was comparable in sham‐operated and BATX rats (Figure 2B_3_). Statistical analyses revealed neither an effect of BAT removal nor a BATX × time interaction for any of the three stimuli (Table S2). Thus, all three stimuli elicited comparable interscapular temperature responses regardless of the presence of interscapular BAT.

### 2.4 Emotional Stress and Immune Stimulation Induce Little or No Expression of Thermogenic Genes in BAT and Minimally Affect BAT Mass

**FIGURE 3:**
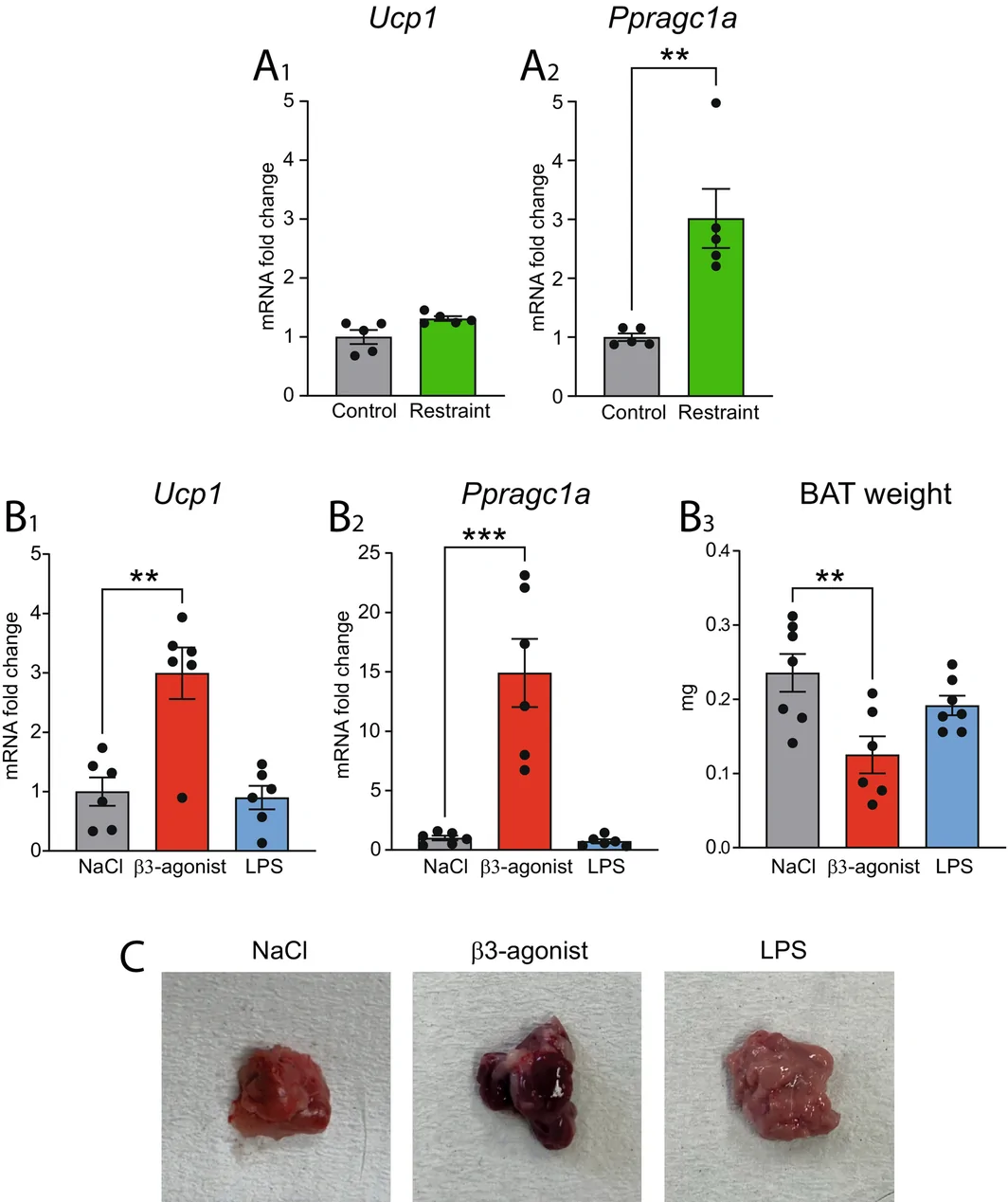
Effects of emotional stress, β_₃_‐adrenergic stimulation, and immune challenge on thermogenic gene expression and BAT morphology in WT rats. (A) Restraint stress. (A_1_) *Ucp1* mRNA expression. (A_2_) *Ppargc1a* mRNA expression. (B) β_₃_‐adrenergic stimulation and immune challenge. (B_1_) *Ucp1* mRNA expression. (B_2_) *Ppargc1a* mRNA expression. (B_3_) BAT weight. One‐way ANOVA: *Ucp1, F*_2,15_ = 14.66, *p* = 0.0003; *Ppargc1a, F*_2,15_ = 23.71, *p* < 0.001; BAT weight, *F*_2,17_ = 6.334, *p* = 0.0088. (C) Representative photographs of BAT following intraperitoneal administration of NaCl, β_₃_‐adrenergic agonist, or LPS, showing marked darkening following β_₃_‐adrenergic stimulation. Error bars indicate SEM. **p < 0.01; ***p < 0.001.

We first examined the effects of restraint stress on thermogenic gene expression in BAT, using naïve rats as controls. As shown in Figure 3A, restraint produced only a minor, statistically nonsignificant increase in *Ucp1* expression. *Ppargc1a*, however, was significantly upregulated, although the increase was considerably smaller than that induced by β₃‐adrenergic stimulation in the immune‐challenge experiment described below.

We next examined the effects of immune challenge. LPS (120 μg kg^−1^, i.p.) did not induce transcription of *Ucp1* or *Ppargc1a*, the latter encoding the transcriptional coactivator PGC‐1α, a key regulator of genes involved in oxidative metabolism [17], relative to saline‐treated controls (Figure 3B_1,2_). LPS also failed to significantly reduce BAT weight (Figure 3B_3_) or alter BAT appearance (Figure 3C). In contrast, administration of the β₃‐adrenergic agonist CL 316243 (1.0 mg kg^−1^, i.p.) robustly induced *Ucp1* and *Ppargc1a* transcription (Figure 3B_1,2_), significantly reduced BAT weight (Figure 3B_3_), and resulted in marked darkening of the BAT (Figure 3C).

The gene‐expression data shown in Figure 3 were normalized to *Gapdh*. Reanalysis using *Tbp* as an independent reference gene similarly showed no induction of *Ucp1* following LPS, with expression instead tending to decrease, while *Ppargc1a* expression was significantly reduced (Figure S4).

We also examined gene expression in wild‐type Sprague–Dawley rats. In this strain, LPS elicited small, statistically nonsignificant increases in *Ucp1* and *Ppargc1a* expression. In contrast, β₃‐agonist administration produced a robust induction of these genes, similar to the response observed in Wistar‐Imamichi rats. Emotional stress induced by cage exchange likewise produced only modest increases in *Ucp1* and *Ppargc1a* expression (Figure S3A,B).

β₃‐agonist treatment significantly reduced BAT weight in Sprague–Dawley rats (one‐way ANOVA: *F*_3,20_ = 4.554, *p* = 0.0137; *p* = 0.0315 vs. saline). By contrast, LPS induced only a small, nonsignificant decrease in BAT weight, and cage exchange produced no detectable change (Figure S3C).

## 3 Discussion

The present study addresses in rats the unresolved question of whether BAT thermogenesis is required for fever and emotionally induced hyperthermia. Although increases in interscapular temperature during immune challenge and emotional stress have been widely interpreted as evidence for activation of BAT [5, 6, 7], this inference has remained untested. Using two complementary approaches—a purpose‐generated UCP1 knockout rat model and surgical removal of interscapular BAT in WT rats—the present study shows that neither UCP1‐mediated thermogenesis nor interscapular BAT is required for the development of immune‐induced fever or psychogenic hyperthermia in rats. These findings do not exclude a contribution of BAT thermogenesis when functional BAT is present, particularly during fever, but demonstrate that both responses can be generated in its absence. Moreover, the increase in interscapular temperature persists both in the absence of UCP1 and following removal of interscapular BAT, demonstrating that the phenomenon widely taken as evidence for BAT activation can occur independently of BAT thermogenesis. Because UCP1 is indispensable for BAT thermogenesis [1, 21], these findings provide direct genetic evidence that neither inflammation‐induced fever nor psychogenic hyperthermia depends on BAT‐mediated heat production. Collectively, our results show that central thermoregulatory circuits need not recruit the same peripheral thermogenic mechanisms during fever as during cold exposure, namely uncoupled mitochondrial respiration in brown adipocytes [15, 22].

This conclusion is particularly relevant in light of the largely indirect evidence that has been taken to support a role for BAT in fever and emotional hyperthermia. Such evidence includes reports of increased sympathetic nerve activity in fibers presumed to innervate BAT [4], as well as observations that interscapular temperature rises before core body temperature under these conditions [5, 6, 7]. We show, consistent with previous observations, that the rise in interscapular temperature indeed precedes the rise in intraperitoneal temperature and, importantly, that the same phenomenon is also observed in UCP1‐deficient animals. The persistence of this response following surgical removal of interscapular BAT further demonstrates that it cannot be attributed to BAT thermogenesis.

These findings indicate that increases in interscapular temperature during fever and emotional stress can arise from mechanisms other than local heat production by BAT. The present data do not identify the effector mechanisms responsible for the increase in body temperature. In fever, reduced heat loss through peripheral vasoconstriction is likely to contribute. In contrast, emotional stress‐induced hyperthermia has previously been shown to occur independently of peripheral vasoconstriction [11], suggesting a greater contribution of heat‐producing mechanisms. Potential contributors include activation of neck and shoulder musculature and increased regional blood flow, both of which accompany emotional stress [23]. Muscle temperature has been shown to rise rapidly in rodents exposed to predator odor [24]. Although skeletal muscle does not express detectable levels of UCP1 [25], it is enriched in UCP3 [26], which does not mediate adaptive thermogenesis [25] but has been implicated in metabolic and redox regulation under conditions of increased energetic demand [27, 28]. Consistent with this, immune challenge increases *Ucp3* transcription in skeletal muscle [11, 27], and deletion of *Ucp3* has been reported to impair febrile responses [10]. Taken together, these observations suggest that skeletal muscle metabolism may contribute to heat production or redistribution, although the underlying mechanisms remain to be established.

The present findings may nevertheless allow reconciliation with earlier reports suggesting BAT involvement. In contrast to our previous mouse studies, in which neither immune stimulation nor emotional stress induced *Ucp1* or *Ppargc1a* transcription [11] (but cf. ref. [29]), Sprague–Dawley rats, but not Wistar–Imamichi rats, displayed a modest induction of *Ucp1* mRNA following lipopolysaccharide administration, and both rat strains showed modest *Ppargc1a* mRNA induction after emotional stress. Interestingly, the increase in *Ppargc1a* expression was not accompanied by induction of *Ucp1*, suggesting that it reflects a more general metabolic response to stress rather than activation of the canonical UCP1‐dependent thermogenic program. In both strains, these transcriptional changes were accompanied by small, statistically nonsignificant reductions in BAT mass after immune challenge, whereas no change was observed following cage exchange.

The interpretation of the gene‐expression data following cage exchange should nevertheless be made with some caution. Because the animals were housed at 22°C–23°C, a temperature at which UCP1 expression is already elevated relative to thermoneutral conditions [11], the absence of *Ucp1* induction does not by itself exclude increased thermogenic activity of preexisting UCP1. In addition, since the hyperthermic response to cage exchange was transient, it remains possible that short‐lived changes in thermogenic gene expression occurred before tissue collection and were therefore not detected. However, a similar pattern of little or no induction of thermogenesis‐related genes was observed following restraint stress, despite the fact that the stressor was maintained continuously for 1 h and tissue was collected shortly thereafter.

Taken together, the present observations suggest that limited UCP1‐dependent BAT activation may occur during fever, although it is clearly not required for the febrile response itself. It remains possible that such auxiliary activation becomes relevant during more prolonged or energetically demanding inflammatory states, when additional heat production may be required [30].

A limitation of the present study is that some experiments were conducted with relatively small sample sizes (*n* = 3–5), reflecting the technically complex and time‐intensive nature of the procedures, including generation of a rat genetic model, implantation of telemetry devices, and surgical removal of interscapular BAT. The observed effects were nevertheless highly consistent across groups, with comparable temperature changes in wild‐type and UCP1‐deficient animals, as well as in animals with intact or removed interscapular BAT, supporting the conclusion that these temperature responses can occur independently of UCP1‐mediated BAT thermogenesis. Moreover, key control experiments confirmed the expected physiological phenotype, including the marked impairment of cold‐ and β_₃_‐adrenergic‐induced thermogenesis in UCP1‐deficient animals. The consistency across these approaches supports the robustness of the conclusions despite the limited sample sizes.

A further limitation is that intraperitoneal and interscapular temperatures were recorded in separate groups of animals rather than simultaneously in the same individuals. As a result, the comparison of temporal dynamics is based on group‐level data. The responses were, however, highly consistent within each group, and the timing of the experimental interventions was precisely controlled, allowing the onset of temperature changes to be determined with minute‐level resolution. The observed differences in temporal dynamics between compartments were therefore robust. Importantly, the conclusion that interscapular temperature can increase independently of BAT thermogenesis is based on genetic and surgical evidence and does not depend on the temporal comparison between compartments.

In summary, our findings demonstrate in rats that fever and emotional stress‐induced hyperthermia do not require UCP1‐mediated BAT thermogenesis, at least under the experimental conditions examined here. Using complementary genetic and surgical approaches, we show that neither UCP1 nor interscapular BAT is required for the elevation of body temperature during immune challenge or emotional stress. These findings challenge both the prevailing view that BAT thermogenesis is an essential effector mechanism underlying these responses and the interpretation that increases in interscapular temperature necessarily reflect BAT thermogenesis. Thus, thermoregulation during immune or emotional stress can rely on peripheral effector mechanisms that differ from those engaged during cold defense.

## 4 Materials and Methods

### 4.1 Animals

Adult Wistar‐Imamichi rats (Imamichi Institute for Animal Reproduction, Tsuchiura, Japan) and Sprague–Dawley rats (Janvier Labs, Le Genest‐Saint‐Isle, France) were used in this study. Animals were housed under controlled environmental conditions (22°C–23°C) with a 14 h light/10 h dark cycle or a 12 h light/12 h dark cycle, respectively, and had *ad libitum* access to food and water.

### 4.2 Ethics Statement

All animal experimental procedures were approved by the Institutional Animal Care and Use Committee of the University of Tokyo and by the Animal Ethics Committee in Linköping and were conducted in accordance with international guidelines for the care and use of laboratory animals.

### 4.3 Generation of UCP1 Knockout Rats

UCP1 knockout (KO) rats were generated using the improved Genome‐editing via Oviductal Nucleic Acids Delivery (i‐GONAD) method [16]. Genome editing was performed with the CRISPR/Cas12 system. The gRNA and Cas12 protein (Alt‐R CRISPR‐Cpf1 crRNA; Alt‐R A.s. CRISPR‐Cas12a (Cpf1) V3) were obtained from Integrated DNA Technologies (Coralville, IA, USA). The target sequence was 5′‐TGATTTCCAGATCCAAGGTGAAGGC‐3′, adjacent to a TTTC PAM site in exon 2.

Female rats were anesthetized with isoflurane (Viatris, Tokyo, Japan) on the day after mating. The prepared genome‐editing solution was injected into the oviductal ampulla using a glass pipette (Narishige, Tokyo, Japan). Electroporation was then performed three times using a CUY21EDIT device (Nepagene, Tokyo, Japan) at 60 V, with a pulse‐on duration of 5.5 ms and a pulse‐off interval of 50.0 ms.

Founder animals were backcrossed to the Wistar–Imamichi strain for two generations before experimental use. Wild‐type littermates served as controls.

### 4.4 Genotyping

Genotyping of offspring was performed by PCR using genomic DNA extracted from tail tissue. PCR reactions were carried out with KOD DNA polymerase (Toyobo, Osaka, Japan). A primer pair, UCP1‐F (5′‐TTATTTAGTCCCCTACCCACCC‐3′) and UCP1‐R (5′‐CTCTTGGACCGTATCGTAGAGG‐3′), was used to distinguish WT (263 bp) and KO (246 bp) alleles. The PCR protocol consisted of an initial denaturation at 94°C for 2 min, followed by 35 cycles at 98°C for 20 s, 60°C for 30 s, and 68°C for 30 s.

PCR products were visualized using a Microchip Electrophoresis System (MultiNA, Shimadzu Corporation, Kyoto, Japan) with the DNA‐500 kit (Shimadzu).

### 4.5 Western Blot Analysis

Proteins isolated from BAT were separated on 8% Tris‐glycine gels, transferred to PVDF membranes, and immunoblotted with a rabbit anti‐UCP1 antibody (1:2500; U6382, MilliporeSigma, Burlington, MA). Signals were detected using a horseradish peroxidase‐conjugated anti‐rabbit IgG antibody and ECL Prime (Amersham Biosciences, Buckinghamshire, UK). β‐Actin was used as a loading control and detected with a mouse anti‐β‐Actin antibody (1:4000; Sigma, St. Louis, MO, USA).

### 4.6 Histology

Interscapular adipose tissue was dissected, fixed in 4% paraformaldehyde, embedded in paraffin, and sectioned at 7 μm. Sections were stained with hematoxylin and eosin and imaged by light microscopy to assess tissue morphology and lipid content.

### 4.7 Surgery

Animals were anesthetized with isoflurane and implanted with a temperature‐sensing and activity‐sensing transponder (E‐Mitter Telemetry System; STARR Life Sciences, Oakmont, PA). Transponders were placed either intraperitoneally to measure core body temperature or subcutaneously in the interscapular region to measure local temperature. After implantation, animals were housed individually.

In a separate series of experiments, interscapular brown adipose tissue (BAT) was surgically removed from wild‐type rats prior to transponder implantation. Sham‐operated wild‐type rats served as controls. All surgical procedures were performed under isoflurane anesthesia under aseptic conditions, and the animals were allowed to recover for 1 week before experimental testing. The interscapular region was shaved and disinfected with 70% ethanol, after which a midline longitudinal skin incision was made. The interscapular BAT pads on both sides were then carefully dissected and removed using fine forceps and scissors. In sham‐operated control animals, the BAT pads were exposed but left intact. Animals were then subjected to cage exchange, restraint stress, or LPS administration as described above.

### 4.8 Cold Stress, Emotional Stress, Immune Challenge, and Adrenergic Activation of BAT

#### 4.8.1 Cold Stress

One week after transponder implantation, animals were exposed to 4°C for 1 week. During the last day, body temperature was monitored by telemetry.

#### 4.8.2 Emotional Stress, Restraint Stress, and Immune Challenge

Emotional stress was induced by transferring rats from their home cage to the cage of another unfamiliar rat (“cage exchange”). Two days later, restraint stress was applied by immobilizing the animals for 1 h in a plastic bag (DecapiCone, Braintree Scientific Inc., Braintree, MA). Four days after restraint stress, rats received an intraperitoneal injection of lipopolysaccharide (LPS; O111: B4; Sigma‐Aldrich, St. Louis, MO; 120 μg kg^−1^) or saline. After a 5‐day washout period, treatments were crossed over such that animals previously given LPS received saline and vice versa.

#### 4.8.3 Adrenergic Activation of BAT

Approximately 1 week after transponder implantation, rats were administered a β_₃_‐adrenergic agonist CL 316243 (1.0 mg kg^−1^ i.p.) or saline.

All injections and stress‐inducing procedures were performed at approximately 10:00 a.m. to minimize circadian variability.

### 4.9 Quantitative Real‐Time PCR

Rats were injected intraperitoneally with LPS (120 μg kg^−1^), the β_₃_‐adrenergic agonist CL 316243 (1.0 mg kg^−1^), or saline, or were subjected to cage exchange or restraint stress as described above. They were euthanized by CO₂ asphyxiation at defined time points: 4 h after LPS injection, 2 h after β_₃_‐agonist injection, 2–3 h after cage exchange, or 1 h after termination of restraint stress.

Interscapular BAT was rapidly excised, placed in RNAlater stabilization reagent (Qiagen, Hilden, Germany), and stored at −70°C until further processing. Total RNA was extracted using the RNeasy Universal Plus kit (Qiagen), and cDNA was synthesized using the High‐Capacity cDNA Reverse Transcription Kit (Applied Biosystems, Foster City, CA).

Quantitative real‐time PCR was performed using TaqMan Gene Expression Master Mix (Applied Biosystems) on a 96‐well plate with a QuantStudio 7 Flex Real‐Time PCR System (Applied Biosystems). The following TaqMan Gene Expression Assays (Thermo Fisher Scientific, Stockholm, Sweden) were used: *Ucp1* (Rn00562126_m1), *Ppargc1a* (PGC‐1α; Rn00580241_m1), and *Gapdh* (Rn01775763_g1).

Gene expression levels were calculated using the comparative Ct (ΔΔCt) method. Target gene expression was normalized to *Gapdh* and expressed relative to the saline‐treated control group. To assess the robustness of the findings, gene‐expression data were also normalized to *Tbp* as an independent reference gene; these analyses are presented in Figure S4.

In addition to the experiments performed in Wistar–Imamichi rats, separate analyses of BAT gene expression and BAT weight following LPS, β_₃_‐adrenergic stimulation, and cage exchange were conducted in Sprague–Dawley rats (Figure S3).

### 4.10 Data Processing and Statistics

Temperature recordings were normalized by subtracting, for each animal, the temperature recorded at 60 min prior to injection/emotional stress, or for analyses of initial response to emotional stress, the value recorded at time point 0.

Statistical analyses were performed using GraphPad Prism version 10 (GraphPad Software, San Diego, CA). Data are presented as mean ± SEM. Temperature responses were analyzed over the response period by two‐way repeated‐measures ANOVA with time as the repeated factor. For comparisons between genotypes or surgical groups, the main effects of group and group × time interactions were evaluated. For comparisons between stimulated and control groups, treatment × time interactions were evaluated. Differences in the slope of temperature increase were examined by linear regression. qPCR data were analyzed using unpaired *t*‐tests or one‐way ANOVA, followed by Tukey's multiple‐comparisons test when appropriate. A *p* < 0.05 was considered statistically significant.

## Author Contributions

**Takashi Matsuwaki:** conceptualization, methodology, investigation, writing – review and editing, writing – original draft, funding acquisition, supervision. **Wakana Harigai:** investigation. **Vladimir Maksimov:** investigation. **Yutaro Nakayama:** investigation. **Kazuyuki Uchida:** investigation. **James K. Chambers:** investigation. **Keitaro Yamanouchi:** investigation. **Anna Eskilsson:** conceptualization, investigation, methodology, writing – review and editing. **Anders Blomqvist:** conceptualization, methodology, funding acquisition, writing – original draft, writing – review and editing, supervision.

## Funding

This work was supported by a Joint Japan‐Sweden (JSPS‐STINT) Research Collaboration grant awarded to Takashi Matsuwaki and Anders Blomqvist (grant numbers JPJSBP120195406 and JA2018‐7623). Takashi Matsuwaki was also supported by JSPS KAKENHI Grants (grant numbers 22H00396 and 23KK0126) and Anders Blomqvist by the Swedish Brain Foundation (grant number FO2025‐0110‐HK‐175).

## Conflicts of Interest

The authors declare no conflicts of interest.

## Supporting information


**Figure S1:** Body weight development in male and female WT, UCP1 KO, and UCP1 heterozygous (Hz) rats. Error bars indicate SEM. *n* = 7–12. Body weight in male KO rats was slightly lower than in wild‐type (WT) or heterozygous (Hz) rats (one‐way ANOVA: *F*_2,24_ = 3.597, *p* = 0.0430; *p* < 0.05 for WT and Hz vs. KO).

**Figure S2:** Core body temperature (upper trace) and locomotor activity (lower trace) during cold exposure (4°C) in three WT and three UCP1 KO rats.

**Figure S3:** Thermogenic gene expression and BAT weight in Sprague–Dawley rats after immune challenge and emotional stress. (A) *Ucp1* mRNA expression. (B) *Ppargc1a* mRNA expression. (C) BAT weight. *n* = 6. One‐way ANOVA: *Ucp1 F*_3,20_ = 18.70, *p* < 0.001; *Ppargc1a F*_3,20_ = 246.6, *p* < 0.001; BAT weight, *F*_3,20_ = 4.554, *p* = 0.0137. Error bars indicate SEM. *p < 0.05; **p < 0.01; ***p < 0.001.

**Figure S4:** Expression of thermogenesis‐related genes normalized to *Tbp*. *Ucp1* and *Ppargc1a* mRNA expression in brown adipose tissue from Wistar‐Imamichi rats following treatment with NaCl, the β_₃_‐adrenergic agonist CL 316243, or LPS. Gene expression was normalized to *Tbp* as an alternative reference gene. Data are presented as fold change relative to the NaCl group. Error bars indicate SEM. *p* < 0.05, *p < 0.01, **p < 0.001.

**Table S1:** Statistical analysis of temperature responses in WT and UCP1 KO rats.

**Table S2:** Statistical analysis of interscapular temperature responses in sham‐operated and BATX rats.

## Data Availability

The data that support the findings of this study are available from the corresponding authors upon reasonable request.
