# Supplementary material for: Immune‐Induced Fever and Psychogenic Hyperthermia Can Occur Independently of Uncoupling Protein 1‐Mediated Brown Adipose Tissue Thermogenesis
Source: Acta Physiol (Oxf). 2026 Aug 25;242(9):e70300. doi: 10.1111/apha.70300 (PMC13505244; doi:10.1111/apha.70300)
Supplement: Supplementary file 1 [file APHA-242-e70300-s001.pdf]

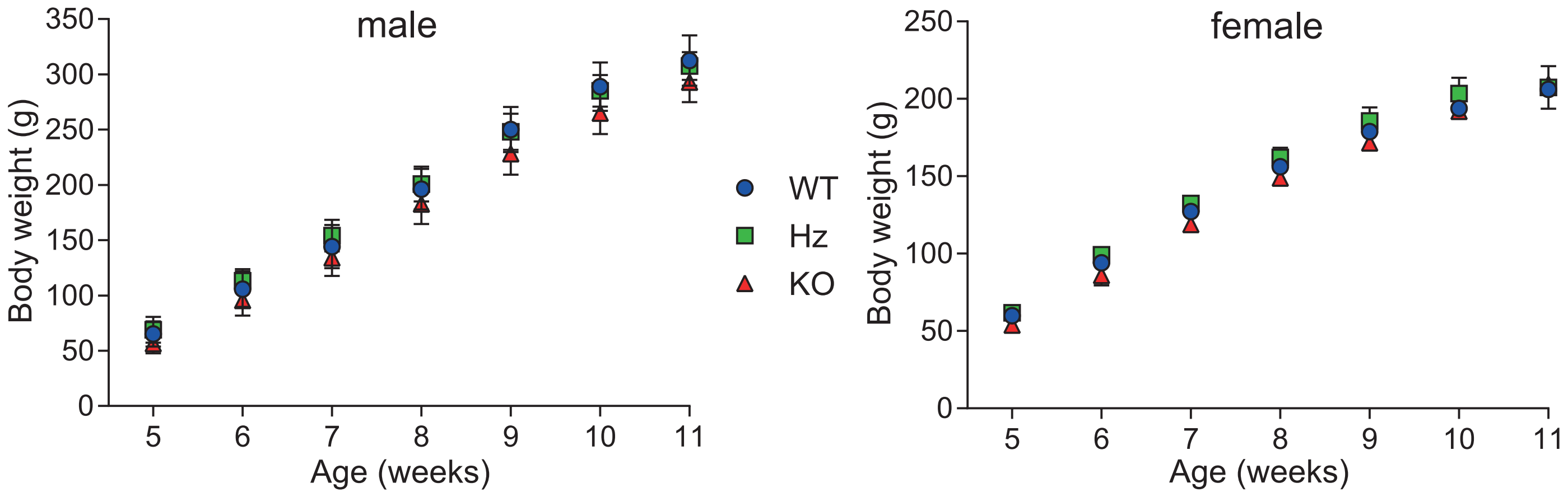

Supplementary Fig. 1. Body weight development in male and female WT, UCP1 KO, and UCP1 heterozygous (Hz) rats. Error bars indicate SEM. n = 7–12. Body weight in male KO rats was slightly lower than in WT or Hz rats (one-way ANOVA: $F_{2,24} = 3.597$, $P = 0.0430$; $P < 0.05$ for WT and Hz vs. KO).
