# Supplementary material for: Immune‐Induced Fever and Psychogenic Hyperthermia Can Occur Independently of Uncoupling Protein 1‐Mediated Brown Adipose Tissue Thermogenesis
Source: Acta Physiol (Oxf). 2026 Aug 25;242(9):e70300. doi: 10.1111/apha.70300 (PMC13505244; doi:10.1111/apha.70300)
Supplement: Supplementary file 2 [file APHA-242-e70300-s003.pdf]

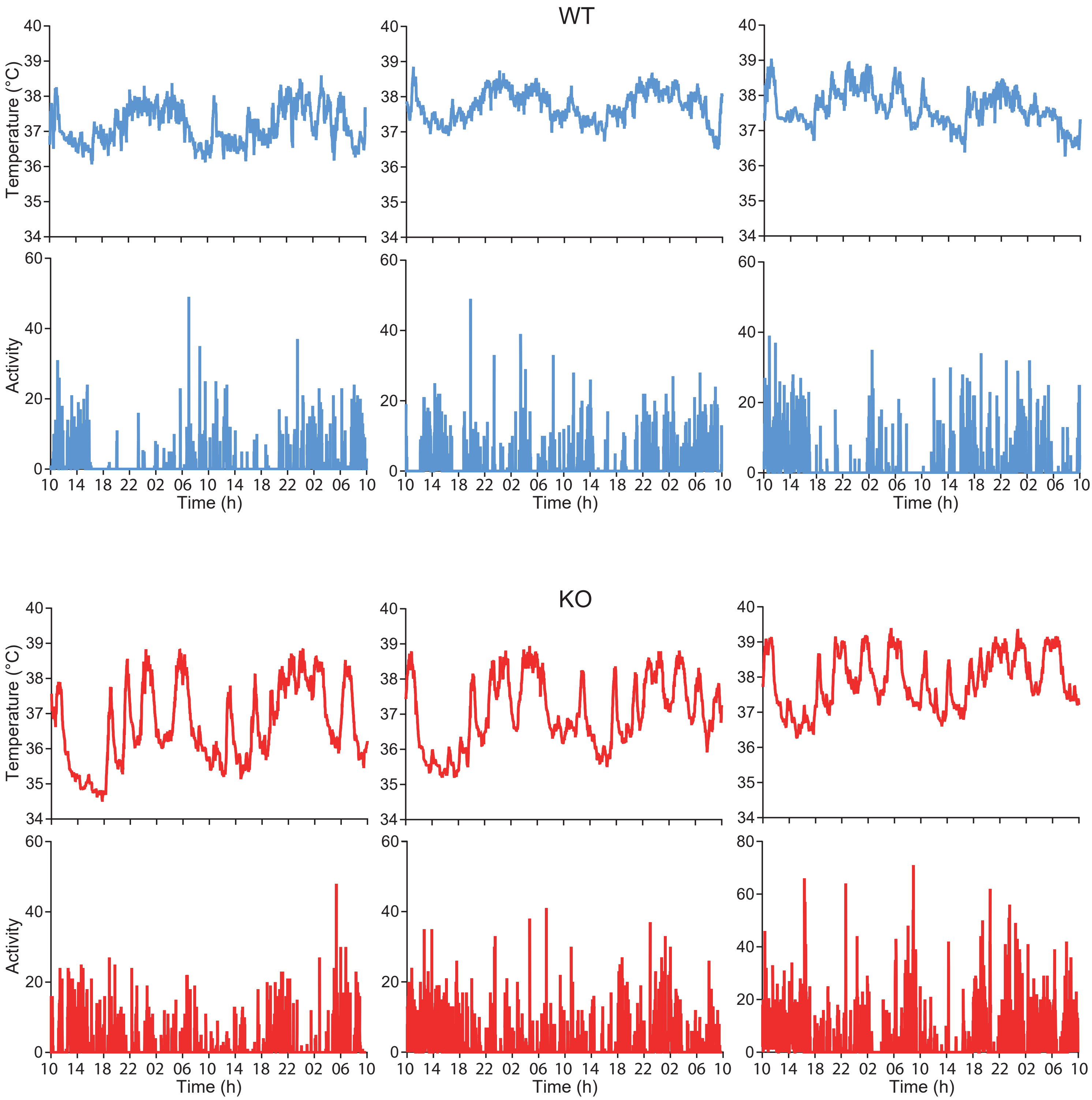

Supplementary Fig. 2. Core body temperature (upper traces) and locomotor activity (lower traces) during cold exposure (4°C) in 3 WT and 3 UCP1 KO rats.
