# Supplementary material for: Immune‐Induced Fever and Psychogenic Hyperthermia Can Occur Independently of Uncoupling Protein 1‐Mediated Brown Adipose Tissue Thermogenesis
Source: Acta Physiol (Oxf). 2026 Aug 25;242(9):e70300. doi: 10.1111/apha.70300 (PMC13505244; doi:10.1111/apha.70300)
Supplement: Supplementary file 3 [file APHA-242-e70300-s005.pdf]

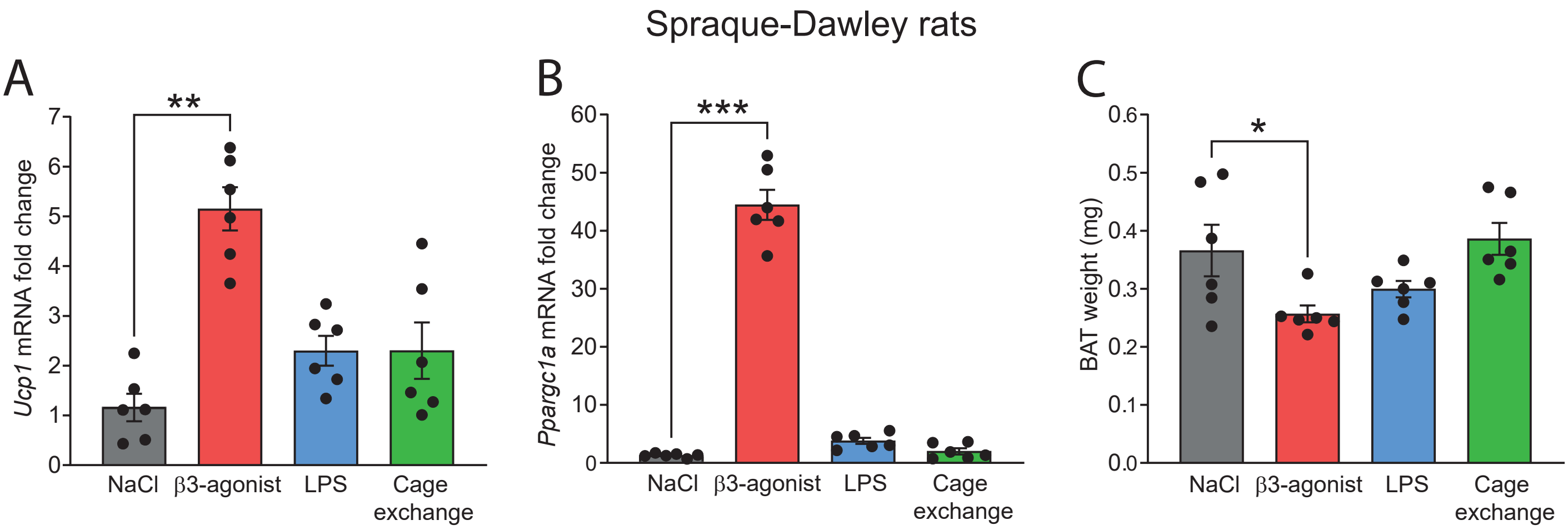

Supplementary Fig. 3. Thermogenic gene expression and BAT weight in Sprague-Dawley rats after immune challenge and emotional stress. (A) *Ucp1* mRNA expression. (B) *Ppargc1a* mRNA expression. (C) BAT weight. n = 6. One-way ANOVA: *Ucp1* $F_{3,20}$ = 18.70, $P < 0.001$; *Ppargc1a* $F_{3,20}$ = 246.6, $P < 0.001$; BAT weight, $F_{3,20}$ = 4.554, $P = 0.0137$. Error bars indicate SEM. *$P < 0.05$; **$P < 0.01$; ***$P < 0.001$.
