# Supplementary material for: Immune‐Induced Fever and Psychogenic Hyperthermia Can Occur Independently of Uncoupling Protein 1‐Mediated Brown Adipose Tissue Thermogenesis
Source: Acta Physiol (Oxf). 2026 Aug 25;242(9):e70300. doi: 10.1111/apha.70300 (PMC13505244; doi:10.1111/apha.70300)
Supplement: Supplementary file 4 [file APHA-242-e70300-s006.pdf]

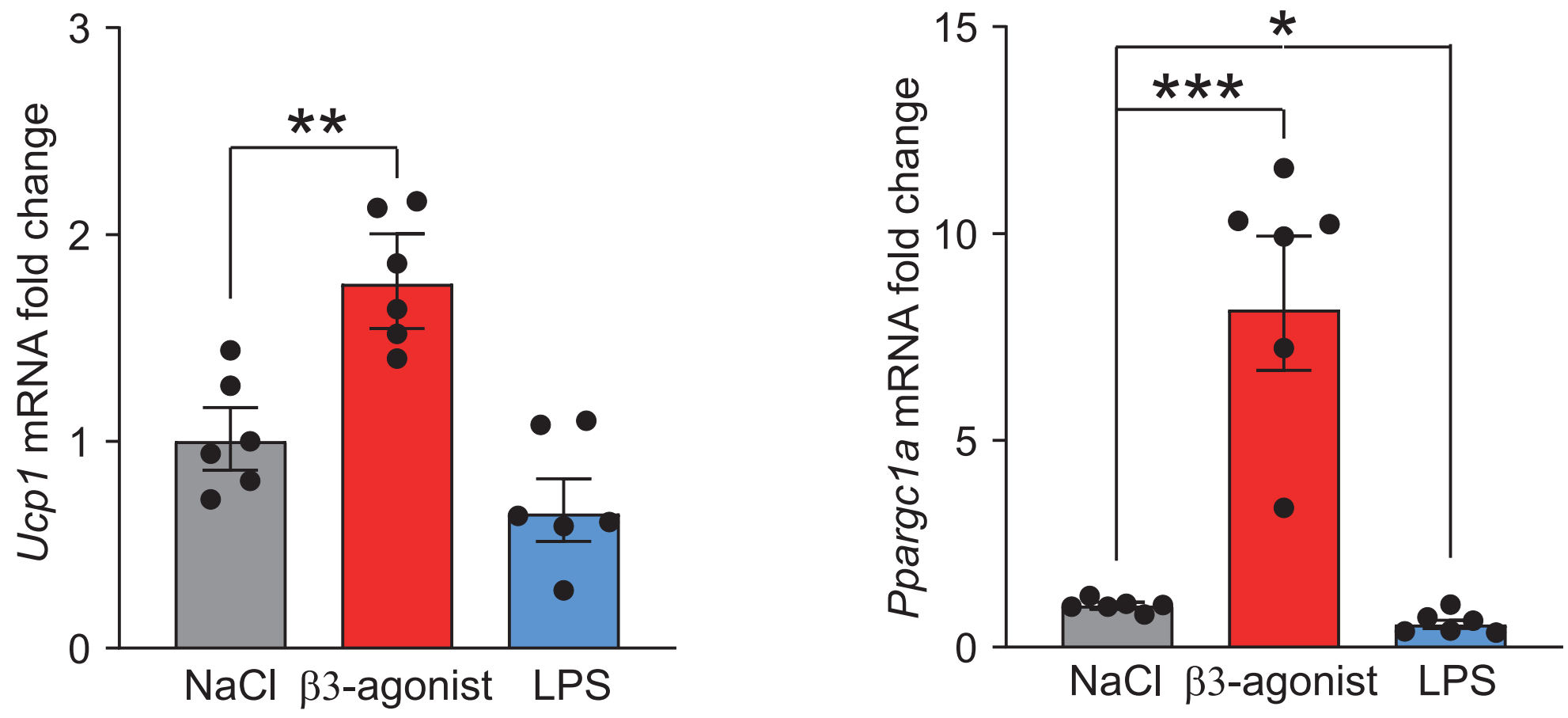

Supplementary Fig. 4. Expression of thermogenesis-related genes normalized to *Tbp*. *Ucp1* and *Ppargc1a* mRNA expression in brown adipose tissue from Wistar-Imamichi rats following treatment with NaCl, the β_3-adrenergic agonist CL 316243, or LPS. Gene expression was normalized to *Tbp* as an alternative reference gene. Data are presented as fold change relative to the NaCl group. Error bars indicate SEM. *$P < 0.05$, **$P < 0.01$, ***$P < 0.001$.
