# Supplementary material for: Immune‐Induced Fever and Psychogenic Hyperthermia Can Occur Independently of Uncoupling Protein 1‐Mediated Brown Adipose Tissue Thermogenesis
Source: Acta Physiol (Oxf). 2026 Aug 25;242(9):e70300. doi: 10.1111/apha.70300 (PMC13505244; doi:10.1111/apha.70300)
Supplement: Supplementary file 5 [file APHA-242-e70300-s002.docx]

# Supplementary Table S2. Statistical analysis of interscapular temperature responses in sham-operated and BATX rats

| Stimulus | Comparison | Effect | F(df1, df2) | P value |
| --- | --- | --- | --- | --- |
| Cage exchange | Sham vs BATX | Surgery × Time | F(59, 354)=0.397 | >0.9999 |
|  |  | Surgery | F(1, 6)=0.018 | 0.8988 |
| Restraint | Sham vs BATX | Surgery × Time | F(119, 714)=1.061 | 0.3237 |
|  |  | Surgery | F(1, 6)=0.437 | 0.5333 |
| LPS | Sham saline vs Sham LPS | Treatment × Time | F(261, 1566)=2.178 | <0.0001 |
|  |  | Treatment | F(1, 6)=11.70 | 0.0141 |
| LPS | BATX saline vs BATX LPS | Treatment × Time | F(261, 1566)=2.559 | <0.0001 |
|  |  | Treatment | F(1, 6)=29.55 | 0.0016 |
| LPS | Sham LPS vs BATX LPS | Surgery × Time | F(261, 1566)=0.794 | 0.9905 |
|  |  | Surgery | F(1, 6)=0.205 | 0.6667 |

Footnote: Two-way repeated-measures ANOVA. Only effects relevant to the experimental hypotheses are shown.
