# Supplementary material for: Immune‐Induced Fever and Psychogenic Hyperthermia Can Occur Independently of Uncoupling Protein 1‐Mediated Brown Adipose Tissue Thermogenesis
Source: Acta Physiol (Oxf). 2026 Aug 25;242(9):e70300. doi: 10.1111/apha.70300 (PMC13505244; doi:10.1111/apha.70300)
Supplement: Supplementary file 6 [file APHA-242-e70300-s004.docx]

# Supplementary Table S1. Statistical analysis of temperature responses in WT and UCP1 KO rats

| Stimulus | Variable | Comparison | Effect | F(df1, df2) | P value |
| --- | --- | --- | --- | --- | --- |
| Cage exchange | Abdominal temperature | WT vs KO | Genotype × Time | F(60, 420)=1.536 | 0.0091 |
|  |  |  | Genotype | F(1, 7)=0.179 | 0.6849 |
| Cage exchange | Interscapular temperature | WT vs KO | Genotype × Time | F(69, 345)=0.515 | 0.9994 |
|  |  |  | Genotype | F(1, 5)=5.637 | 0.0636 |
| Restraint | Abdominal temperature | WT vs KO | Genotype × Time | F(90, 630)=0.483 | >0.9999 |
|  |  |  | Genotype | F(1, 7)=0.256 | 0.6286 |
| Restraint | Interscapular temperature | WT vs KO | Genotype × Time | F(60, 300)=0.285 | >0.9999 |
|  |  |  | Genotype | F(1, 5)=0.356 | 0.5768 |
| LPS | Abdominal temperature | WT saline vs WT LPS | Treatment × Time | F(450, 2250)=5.248 | <0.0001 |
|  |  |  | Treatment | F(1, 5)=20.82 | 0.0060 |
| LPS | Abdominal temperature | KO saline vs KO LPS | Treatment × Time | F(450, 2700)=3.138 | <0.0001 |
|  |  |  | Treatment | F(1, 6)=14.15 | 0.0094 |
| LPS | Abdominal temperature | WT LPS vs KO LPS | Genotype × Time | F(450, 2700)=1.131 | 0.0401 |
|  |  |  | Genotype | F(1, 6)=2.737 | 0.1491 |
| LPS | Interscapular temperature | WT saline vs WT LPS | Treatment × Time | F(155, 2170)=8.115 | <0.0001 |
|  |  |  | Treatment | F(1, 14)=1.717 | 0.2111 |
| LPS | Interscapular temperature | KO saline vs KO LPS | Treatment × Time | F(155, 1705)=4.467 | <0.0001 |
|  |  |  | Treatment | F(1, 11)=2.236 | 0.1629 |
| LPS | Interscapular temperature | WT LPS vs KO LPS | Genotype × Time | F(155, 2015)=1.090 | 0.2193 |
|  |  |  | Genotype | F(1, 13)=2.45×10⁻⁵ | 0.9961 |

Footnote: Two-way repeated-measures ANOVA or a mixed-effects model (REML). Only effects relevant to the experimental hypotheses are shown.
